# Effect of Teachers’ Happiness on Teachers’ Health. The Mediating Role of Happiness at Work

**DOI:** 10.3389/fpsyg.2019.02449

**Published:** 2019-10-31

**Authors:** Paula Benevene, Simona De Stasio, Caterina Fiorilli, Ilaria Buonomo, Benedetta Ragni, Juan José Maldonado Briegas, Daniela Barni

**Affiliations:** ^1^Department of Human Studies, Libera Università Maria SS. Assunta, Rome, Italy; ^2^Department of Business Management and Sociology, University of Extremadura, Badajoz, Spain

**Keywords:** teachers’ happiness, teachers’ health, happiness, self-esteem, happiness at work, happiness at school, subjective happiness

## Abstract

The present study aims to expand the understanding of the effects of dispositional happiness and self-esteem, as dispositional traits, on the health of teachers, as well as to understand the role played by the working environment in generating positive affection, thus mediating between the dispositional traits and teachers’ health. Two hundred and eighty-two full-time in-service teachers (93.6% female) from Rome (Italy) took part in this study. Their ages ranged from 26 to 55 (*M* = 40.49 years, *SD* = 5.93). Participants’ teaching experience ranged from 1 to 31 years (*M* = 9.95 years, *SD* = 5.65). 30.6% of participants taught in kindergarten (for children aged 0–5 years), 42.6% in primary schools (for children aged 6–11 years), 15.8% in middle schools and 10.9% in high schools. A questionnaire was administered, containing: the Subjective Happiness Scale (SHS); the Rosenberg Self-Esteem Scale (RSES); The adapted version for teachers of the School Children Happiness Inventory (Ivens, 2007); the Physical and Mental Health Scales (SF12). The data were analyzed using the MPLUS software, version 8. Our results showed that teacher happiness at work partially mediates the relationship between dispositional happiness and teacher health, and fully mediates the relationship between self-esteem and teacher health. To the best of our knowledge, the mediational role of teacher happiness has not been addressed before, concerning these dimensions. At the same time, our findings confirmed the role of self-esteem in endorsing health-related behaviors, thus promoting physical and mental health. Moreover, according to our study findings, when teachers acknowledge their workplace as a context in which they feel happy, the impact of dispositional happiness and self-esteem on health conditions is higher. Effective measures to promote teachers’ well-being are discussed.

## Introduction

Teachers’ health has received much more attention over the past century (Skaalvik and Skaalvik, 2011), since schoolteachers have emerged as a category of workers highly prone to a variety of psychological, mental and physical problems, as a consequence of the stress and attrition they are dealing with every day (Borrelli et al., 2014; Benevene and Fiorilli, 2015). Consistently, there is extensive academic literature showing that stress is one of the leading causes of teachers’ sickness absence, ill-health retirement and turnover (Fiorilli et al., 2015). High rates of teachers’ attrition are found both among Western and non-Western countries, notwithstanding the relevant cultural, organizational and educational differences across the countries (Chang, 2009; Hong, 2010; Fiorilli et al., 2015). On the other hand, the approach of the positive psychology has shown that dispositional traits have an impact on how teachers, and more in general, individuals, successfully manage the challenges they have to face in their workplace and cope with stressful events (Xanthopoulou et al., 2007; De Stasio et al., 2017). It is also a fact that happiness in the workplace is often influenced by one’s own working experiences events. There is evidence that individuals may experience a higher level of happiness than usual days, compared with their baselines, when they feel fulfilled in their basic needs for competence, autonomy and relatedness in major activities in their workplace (Sheldon et al., 1996; Reis et al., 2000; Buonomo et al., 2019). It has to be noted that happiness at work has been mainly approached in relation to the effects of the characteristics of the organization or the job on individuals, thus devoting little attention to the cognitive and affective processes. From these studies, it has emerged that the main factors leading to a higher degree of happiness at work are: positive interpersonal relationship, both with colleagues and superiors (Macuka et al., 2017); performing activities perceived as meaningful and fruitful; recognition for the achievements (Fisher, 2010), fair treatment (Chaiprasit and Santidhiraku, 2011) and a positive family and work-life balance (Golden et al., 2013). On the other hand, the effect of the stable attributes of individuals on happiness at work has recently been recognized, thus showing the need for deepening the knowledge on how dispositional, psychological variables are linked to job happiness (Fisher, 2010; Warr, 2014). Namely, individual characteristics identified as antecedent happiness in the workplace are: personality traits such as extraversion, responsibility and agreeability (Warr, 2014), as well as positive dispositional affect (Fisher, 2010; Seligman, 2011).

The present study aims to deepen the understanding of the effect of dispositional happiness and self-esteem, as dispositional traits, on the health of a group of teachers, as well as the understanding of the role played by the working environment in generating positive affection, thus mediating between the dispositional traits and teachers’ health. Teachers represent a group of employees that is still poorly addressed by the literature in this regard, therefore, addressing this topic might offer the opportunity for identifying effective measures to promote teachers’ well-being (Fisher, 2010; Kok et al., 2013).

### Happiness as a Dispositional Trait Linked to Teachers’ Health

Happiness is probably the dispositional trait most studied in relation to individuals’ health (Fredrickson and Levenson, 1998), despite the paucity of studies carried out among teachers. The construct of dispositional, subjective happiness, refers the global judgment of oneself as a happy or an unhappy person, which tends to be rather stable across years, in spite of life changes and daily routines (Diener et al., 1997; Lyubomirsky and Lepper, 1999; Lucas, 2007; Luhmann et al., 2012).

A considerable number of studies have proven the protective role in facing life challenges and therefore generating positive health outcomes, such as subjectively perceived health, disease rates and severity, as well as mortality. For instance, the effect of happiness on longevity among the healthy population is similar to that of smoking or not, while negative emotions are often predictors of worse health outcomes (Diener and Seligman, 2004; Veenhoven, 2008). Dispositional happiness protects individuals’ health at least in three ways. Firstly, chronic unhappiness activates the flight-flight response, which is the physiological reaction that occurs in response to a perceived harmful event, attack, or threat to survival. This activation in the long run is related to harmful effects, such as higher blood pressure and a lower immune response. Furthermore, studies carried out in the field of psychosomatic medicine have proven the protective effects of positive mental states against illness, since they enhance better immune response (Cohen et al., 1995; Robinson et al., 2012). Secondly, happy people are more inclined to live healthier. Happy people tend to watch their weight, do not exceed in smoking and drinking, and engage in sports. Moreover, they are quicker at identifying symptoms of illness and more successful in coping with threatening information (Graham, 2008; Angner et al., 2009; Van Cappellen et al., 2018; Steptoe, 2019). Thirdly, happiness may promote health through the development of a more extensive repertoire of positive actions to cope with life events (Zautra, 2006). Happy people are more able to develop and maintain supportive social networks and to make better choices in life, since “they are more open to the world and more self-confident. they are also less likely to fall victim to the pattern of one-dimensional thinking in distress, which might hamper choice” (Veenhoven, 2008, p. 459).

The effects of happiness on individuals’ health may be explained in the light of the Fredrickson’s (2013) broaden-and-build theory, which posits that positive affect helps to ‘build’ resources that are effective in creating healthier living conditions. More specifically, “certain discrete positive emotions—including joy, interest, contentment, pride, and love—although phenomenologically distinct, all share the ability to broaden people’s momentary thought-action repertoires and build their enduring personal resources, ranging from physical and intellectual resources to social and psychological resources.” (Fredrickson, 2001, p. 220). Thus, experiencing positive emotions tends to expand the spectrum of the options to be considered; conversely, negative emotion may narrow the strategies available to the mind. Moreover, the resources generated by positive emotions last over time, and therefore can be retained for future use, in different emotional states (Fredrickson and Branigan, 2005). As far as the school is considered as a workplace, the paucity of previous studies carried out on the subjective happiness and health among teachers, belonging to different countries and educational systems, has confirmed the link between these two constructs. Namely, from a comparative study, carried out in Hong Kong and Italy among two groups of kindergarten teachers, it emerged a negative correlation of self-esteem and subjective well-being with mental health complaints. Another study carried out in India, among K-12 teachers in the state of Kerala, proved the correlation of self-esteem and subjective happiness on teachers’ health (Benevene et al., 2018a, b). However, no previous study has addressed the mediating role of positive affect at works between dispositional happiness and teachers’ health. The relevance of deepening the knowledge about this dynamic lies in the opportunities that increased awareness of this subject offers, in terms of preventing teachers’ illness and enhancing their health, through training and positive school management.

Therefore, the following hypothesis was formulated:

H1. Teachers’ subjective happiness is related to teachers’ health.

### Self-Esteem as a Dispositional Trait Linked to Teachers’ Health

Like subjective happiness, self-esteem is also a dispositional trait that highly contributes to individuals’ health and quality of life (Antonucci and Jackson, 1983; Evans, 1997). Unsurprisingly, there are pieces of evidence that self-esteem is highly linked with happiness (Hornsey et al., 2018). Self-esteem is described as the evaluative and affective dimension of self-concept, as it refers to the global appraisal of one’s overall positive or negative value (Brown and Marshall, 2001; Leary and Baumeister, 2004; Rosenberg et al., 2006).

Self-esteem operates as a protective factor, buffering against the impact of adverse life events. There is evidence that it actively promotes the healthy functioning of individuals in managing their life aspects, generating positive social behavior, achievements, satisfaction, fulfillment, as well as the ability to cope with diseases like cancer and heart disease (Baumeister et al., 2003). Conversely, people with low self-esteem are at a high risk of developing depression, burnout, anorexia nervosa, bulimia, anxiety, violence, substance abuse and high-risk behaviors (Schimmack and Diener, 2003; Schmitz et al., 2003; Collie et al., 2012; Sowislo and Orth, 2013). The protective nature of self-esteem has become evident through the studies where chronically ill individuals were observed. They showed that high self-esteem, in combination with enjoying close relationships, directly protects individuals from developing depressive symptoms (Penninx et al., 1998; Prince et al., 2007). From these studies, it emerged that self-esteem prevents from developing stress or physical disease in situations where individuals might experience fear and uncertainty (Mann et al., 2004). More in general, self-esteem has been addressed by many studies on stress and coping, demonstrating how this factor buffers the impact of stressors. In fact, according to the transactional model of stress and coping developed by Lazarus and Folkman (1984), high self-esteem mitigates the perceived threats and improves the quality of the coping strategies. As Lazarus and Folkman state: “Viewing yourself positively can also be regarded as an essential psychological resource for coping. We include in this category those general and specific beliefs that serve as a basis for hope and that sustain coping efforts in the face of the most adverse condition” (Lazarus and Folkman, 1984, p. 159). Thus, self-esteem can be seen as an internal moderator of stressors (Mann et al., 2004) and, more in general, as a crucial component of individual adaptability, thereby enabling resilience in facing difficulties (Hobfoll, 2002; Betoret, 2006; Tsouloupas et al., 2010). Unsurprisingly, from many studies it has arisen that self-esteem is a crucial resource for coping with the challenges of teaching, playing a leading role in teachers’ health (Mäkikangas et al., 2004; Benevene et al., 2018a). Conversely, low self-esteem is associated with mental health problems, such as depression and burnout (Roberts and Kendler, 1999; Schimmack and Diener, 2003; Collie et al., 2012).

Therefore, the following hypothesis was formulated:

H2. Teachers’ self-esteem is related to teachers’ health.

### The Mediating Role of Happiness at Work Between Dispositional Traits and Teachers’ Health

Despite their dispositional traits, teachers have to face and cope with specific working conditions, which might result in either challenging or enhancing their happiness. Teachers do not operate in a vacuum and schools are an emotional arena, able to elicit positive or negative feelings in the interaction of individuals with their working environment. As Fineman (2012) noted, teaching experience may generate significant and repeated unpleasant emotions, eventually leading to high level of stress and negative consequence on the general state of health. In this regard, the transactional theory of Lazarus and Folkman (1984) posits the dynamic, mutually reciprocal, bidirectional interactions of teachers with their workplace, including factors such as teacher perceived leadership style, attribution of student misbehaviors, and perceived exchange of investments and outcomes (Bibou-Nakou et al., 1999; Friedman, 2000; Evers et al., 2004; Van Horn et al., 2004). This theory underlines the cognitive process through which individuals mold their interaction with the specific characteristics of their environment, in the light of their assessment of the stimuli and their coping strategies. Therefore, happiness and self-esteem, as dispositional resources, not only impact individuals’ health, but also how individuals respond to their working environment, activating further resources to face the challenges of their workplace (Sutton et al., 2009). There is evidence to prove that people with positive thoughts are healthier and more successful in their working life; they are more productive and efficient, generating a competitive advantage for their organizations (Momeni et al., 2011). Therefore, it can be hypothesized that happiness and self-esteem, as dispositional traits, may be linked with happiness in the workplace, namely teachers’ happiness at work. On the other hand, the interaction between individuals and their working environment, as the transactional theory, also suggests the workplace may elicit positive or negative feelings and emotions, therefore generating positive or negative affect at work, which in turn has an effect on teachers’ health (Argyle, 1997; Fredrickson, 2000). In fact, positive thoughts at work help individuals to enjoy a healthier life (Raei Dehaghi, 2012). Unsurprisingly, the impact of happiness on the health of employees is strictly linked with low levels of absenteeism and sick leave and high performance (Peterson and Park, 2011).

Therefore, the following hypotheses were formulated:

H3. Teachers’ happiness at work plays a mediation role between self-esteem and teachers’ health.
H4. Teachers’ happiness at work plays a mediation role between dispositional happiness and teachers’ health.

At the same time, acknowledging the multiple theories that account for relationships between positive emotions and self-esteem, which differ from the ones considered in our study, will allow for considering alternative associations among our predictive and mediating variables, as described in the data analysis strategy. More specifically, while the measured variables are suitable to verify the hypothesis that self-esteem is a predictor of happiness (e.g., Ford et al., 2016), they are not suitable to compare our model with others, considering more complex relationships (e.g., Alessandri et al., 2012; Barbaranelli et al., 2019).

## Materials and Methods

### Participants and Procedure

Two hundred and eighty-two full-time in-service teachers (93.6% female) from Rome (Italy) took part in this study. Their ages ranged from 26 to 55 (*M* = 40.49 years, *SD* = 5.93). Regarding demographic variables (marital status and children), 54.7% of teachers had a partner, 38.1% were single, 6.8% were separated/divorced, while only one teacher was widowed. Furthermore, sixty-four percent of participants had children. Regarding job-related dimensions, participants’ teaching years of experience ranged from 1 to 31 (*M* = 9.95, *SD* = 5.65). Teachers’ age and years of experience are positively correlated (Pearson’s *r* = 0.365, *p* = 0.000). 30.6% of participants taught in kindergartens (for children aged 0–5 years), 42.6% in primary schools (for children aged 6–11 years), 15.8% in middle schools and 10.9% in high schools. Thus, the teachers involved constitute a convenience sample, which cannot be considered as being representative of the entire population of Italian teachers. Data were gathered by the research group at the end of school board meetings, on a voluntary basis in an individual setting. The entire process was anonymous. Participants took part to the study after having received written information on Italian privacy regulations and having signed informed consent. The research was conducted following the APA’s ethical principles and code of conduct (American Psychological Association, 2016). When an Italian validation was not available, the original versions of questionnaires were initially translated from English into Italian and then back-translated into English to check the alignment with the original versions.

### Measures

The Subjective Happiness Scale (SHS; Lyubomirsky and Lepper, 1999; Italian version by Iani et al., 2014) is a 4-item scale aimed at assessing subjective happiness, using a 7-point Likert scale. The first two items ask people to rate how they happy they are about their life in general (1 = *not a very happy person*, 7 = *a very happy person*) and how happy they are in comparison with their peers (1 = *less happy*, 7 = *happier*); the last two items ask respondents to what extent the characterization of a happy and an unhappy person describe themselves (1 = *not at all*, 7 = *a great deal*). Higher scores on this measure indicate higher subjective happiness. Cronbach’s alpha was 0.81.

The Rosenberg Self-Esteem Scale (RSES, Rosenberg, 1965; Italian version by Prezza et al., 1997) consists of 10 items and assesses self-esteem, with a 4-point Likert scale (1 = I *strongly disagree*, 4 = *I strongly agree*). Higher scores indicate higher self-esteem. Scores between 15 and 25 are within normal range; scores below 15 suggest low self-esteem. Cronbach’s alpha was 0.81.

Teacher Happiness at work was evaluated with the adapted version for teachers of the School Children Happiness Inventory (Ivens, 2007). The adapted version consists of 33 items, measured on a 4-point Likert scale (1 = *I strongly disagree*, 4 = *I strongly agree*). Higher scores indicate high perceived happiness at school. The adapted version, despite not being validated yet, showed good reliability (Cronbach’s alpha 0.93).

The 12-item SF Health Survey (SF12; Ware et al., 1996; Italian version by Kodraliu et al., 2001) is a short survey composed of 12 questions, selected from the SF-36 Health Survey (Ware et al., 1996). Items are grouped into two sub-scales: Physical health (PH, 6 items), assessing limitations in physical activity and functioning, pain and overall health; Mental health (MH, 6 items) assessing mental health, vitality, and social functioning. The scale comprehends items assessed with dichotomous questions, as well as measured with 3- to 6-point Likert scales. Overall, the score ranges from 0 to 100, where higher scores represent better health. Cronbach’s alpha was 0.83.

### Data Analysis

First, a Confirmatory Factor Analysis (CFA, Kline, 2015) was performed in order to examine the measurement model with MPlus version 8 (Muthén and Muthén, 2017).

To enhance the reliability and parsimony of our model, item parcels were created for self-esteem measure (10 items) and teacher happiness at work measure (33 items). Each factor was defined by three parcels, to obtain less free parameters to estimate and to reduce the sources of sampling error (Little et al., 2002, 2013; Coffman and MacCallum, 2005), and each parcel was created by sequentially summing items assigned based on the highest to lowest item-total corrected correlations (Little et al., 2002, 2013; Coffman and MacCallum, 2005). The Robust Maximum Likelihood Approach (MLR) was used to deal with non-normality in data (Wang and Wang, 2012).

Next, the structural model (Model 1) was tested by using the structural equation modeling (SEM) approach (Kline, 2011). The model was conceptualized by using subjective happiness (as measured by SHS), self-esteem (as measured by RSES), happiness at work (as measured by the adapted version of TSCHI), and health (as operationalized by physical and mental health). We hypothesized both direct and indirect (through teachers’ happiness at work) effects of subjective wellbeing and self-esteem on health. Moreover, we tested an alternative model (Model 2) in order to investigate whether a model, in which dispositional and work-related happiness mediated the effect of self-esteem, better predicted health outcomes than a model in which self-esteem and subjective happiness were considered as dispositional variables.

In order to compare the two models, given that Model 2 was non-nested within our hypothesized model, we used three information criteria: AIC, BIC and Sample-Size Adjusted BIC. Lower values of these indices indicate a better model (Wang and Wang, 2012).

The following procedures of data exploration were applied: (a) uni- and multivariate outlier analysis (Mahalanobis’s distance was set to *p* < 0.001, Gath and Hayes, 2006); (b) score distribution analysis (skewness and kurtosis cut-off points were set to [−2; +2] (George and Mallery, 2003); (c) missing value analyses (missing values were skipped listwise, Little, 1992). At the end of these procedures, we obtained the sample described above.

## Results

### Measurement Model

The measurement model showed a good fit to the data: χ2(48) = 66.974, *p* = 0.036, CFI = 0.977, TLI = 0.968, RMSEA = 0.056 (90% CI = 0.015–0.086), *p* = 0.357, SRMR = 0.054., confirming validity and distinguishability of the four theoretical constructs.

The correlations among the studied variables are presented in Table 1. Teacher happiness at work was significantly correlated with teachers’ subjective happiness (*r* = 0.48), perceived self-esteem (*r* = 0.47) and health (*r* = 0.63). Moreover, teachers’ health was significantly correlated with teachers’ subjective happiness (*r* = 0.47) and perceived self-esteem (*r* = 0.40). Sociodemographic and job-related variables are not shown, as their associations with the variables of interest are not significant.

**TABLE 1:** Bivariate correlations between teachers’ variables.

	**1**	**2**	**3**	**4**
1. Subjective happiness	1			
2. Self-esteem	0.55^∗∗^	1		
3. Teacher happiness at work	0.48^∗∗^	0.47^∗∗^	1	
4. Teacher health	0.47^∗∗^	0.40^∗∗^	0.63^∗∗^	1

*^∗∗^*p* < 0.01.*

### Final Model

**FIGURE 1:**
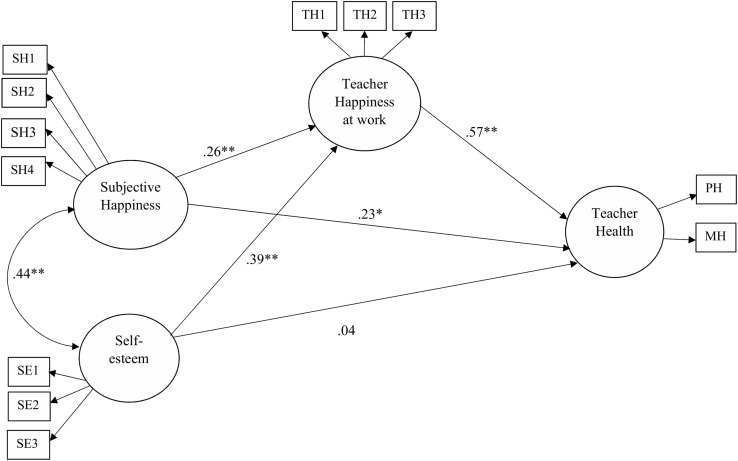
Results of the structural equation model. Standardized direct effects were reported. ^∗^*p* < 0.05, ^∗∗^*p* < 0.01. PH, physical health; MH, mental health.

Model 1 (Figure 1), hypothesizing both direct and indirect (through teachers’ happiness at work) effects of subjective happiness and self-esteem on health, proved to be an adequate fit to the data: χ2(48) = 66.974, *p* = 0.036, CFI = 0.977, TLI = 0.968, RMSEA = 0.056 (90% CI = 0.015–0.086), *p* = 0.357, SRMR = 0.054. Overall, subjective happiness was associated with both teachers’ health (*b* = 0.23, *p* = 0.029) and teachers’ positive affects at work (*b* = 0.390, *p* = 0.000). Furthermore, self-esteem resulted associated with teachers’ positive affects at work (*b* = 0.25, *p* = 0.005), while the association between self-esteem with teachers’ health was not statistically significant. Finally, teachers’ happiness at work showed a significant direct effect on teachers’ health (*b* = 0.57, *p* = 0.000). The percentages of variance explained were 53.6% for teachers’ health and 30.6% for teachers’ happiness at work. Subjective happiness and self-esteem were positively correlated (*r* = 0.44, *p* = 0.000).

In line with our hypothesis (H3 and H4), teachers’ happiness at work mediated the effects of both subjective happiness and self-esteem on teachers’ health. Specifically, teachers’ happiness at work partially mediated the effects of subjective happiness on health (*b*_DIRECT_ = 0.23, *p* = 0.029; *b*_INDIRECT_ = 0.22, *p* = 0.001; total indirect effect:0.45, *p* = 0.000) and fully mediated the effect of self-esteem on teachers’ health (*b*_DIRECT_ = ns.; *b*_INDIRECT_ = 0.15, *p* = 0.021; total indirect effect: 0.18, *p* = 0.041; Hayes, 2013).

The alternative model (Model 2) examined both direct and indirect (through teachers’ happiness at work and subjective happiness) effects of self-esteem on health. The model showed a poor fit to the data χ2(49) = 82.169, *p* = 0.002, CFI = 0.960, TLI = 0.946, RMSEA = 0.073 (90% CI = 0.044–0.100), *p* = 0.087, SRMR = 0.101. Moreover, comparing the two models, Model 1 showed lower values on the three information criteria (AIC = 5803.565, BIC = 5922.689, Sample-Size Adjusted BIC = 5789.872) than Model 2 (AIC = 5817.272, BIC = 5933.560, Sample-Size Adjusted BIC = 5803.905). For these reasons we concluded that Model 2 could be rejected, and we retained Model 1 as the final model of the study.

## Discussion

Our findings show that teacher happiness at work partially mediates the relationship between dispositional happiness and teacher health, and fully mediates the relationship between self-esteem and teacher health. To the best of our knowledge, the mediational role of teacher happiness has not been addressed before with regard to these dimensions.

With regard to the role of dispositional happiness and self-esteem in predicting health, we confirmed, in a sample of Italian teachers, that individuals who tend to experience positive emotions about themselves and their life events are more likely to build healthy life conditions (Fredrickson, 2001; Pressman et al., 2018). Our results confirmed the broaden and build theory (Fredrickson, 2001), which assumes that experiencing and acknowledging positive emotions leads to several positive outcomes, in terms of physical (Fredrickson et al., 2000; Gloria et al., 2013) and mental health (Tugade and Fredrickson, 2007; Tugade, 2011), and, more generally, in terms of positive self-perceptions (Gloria et al., 2013). According to Pressman et al. (2018), in fact, acknowledging positive affects heightens the likelihood of pursuing positive health behaviors, and influences the patterns of physiological arousal, thus leading to better health outcomes. At the same time, our findings confirmed the role of self-esteem in endorsing health-related behaviors (Mäkikangas et al., 2004), thus promoting physical and mental health (Mann et al., 2004; Macinnes, 2006; Benevene et al., 2018a), despite only with the mediation of the workplace context.

At the same time, findings of our study shed light on the role of happiness at work. More specifically, it emerged that the impact of dispositional happiness and self-esteem on health conditions is higher when teachers acknowledge their workplace as a context in which they feel happy. This result is consistent with Lyubomirsky’s model of hedonic adaptation (Lyubomirsky, 2012; Dutt et al., 2013). According to this author, being prone to happiness is not enough when it comes to prolonging the positive effects of happiness over time. The hedonic adaptation effect suggests that people tend to get used to happiness and positive emotions, unless they experience more variety and frequency of positive events in their life. Consistently, the teaching profession includes several dimensions: didactical, pedagogical, caring, relational (Beijaard et al., 2000; O’Connor, 2008; Hanna et al., 2019). Furthermore, the ways teachers perceive these dimensions differs according to personal history, teaching context, professional experiences, and so on (e.g., Beijaard et al., 2000; Buonomo et al., 2017; Fiorilli et al., 2017). These findings suggest that teachers, considering the heterogeneous nature of their profession, may experience a variety of duties, relationships and tasks during their daily job. This is confirmed by studies about organizational citizenship behaviors and extra-mansions tasks in teachers (Somech and Drach-Zahavy, 2000; Belogolovsky and Somech, 2010). Thus, our results could be explained by Lyubomirsky’s theory about the need to live in social contexts perceived as various and stimulating.

Moreover, Bao and Lyubomirsky (2014) stated that positive experiences occurring in contexts in which individuals pursue intrinsic, self-determined goals boost the effects of positive experiences in the long term. Several studies have shown that teachers are prone to motivate their professional choice based on personal and social utility values, considering teaching as a mission and, thus, a way to express themselves (Schwarz, 1999; Watt and Richardson, 2007; Fokkens-Bruinsma and Canrinus, 2012).

Consistently, Pressman et al., 2018 stated that the influence of positive affect on health is partially mediated by individuals’ social, intellectual, psychological and physical resources. With this regard, other authors claim that positive workplaces support these resources, as they are inherently interesting, empowering and supportive for employees (Henry, 2015). Therefore, it is likely that the teachers who recognize their workplaces as sources of happiness, have higher chances to feel healthy. Previous studies have included several job-related sources of happiness in teachers: students’ achievement and recognition, positive management of work demands, colleagues’ and principal’s support (Hargreaves, 2000; Tadić et al., 2013; Borrelli et al., 2014). Consistently, interventions aiming at promoting positive workplaces usually address job enhancement, intrinsic motivation, flexible working conditions, social exchanges, positive recognition, and participation (Henry, 2015). Living in such workplaces, in turn, promotes workers’ happiness and physical health (Bono et al., 2013; Salanova et al., 2013; Gilbert et al., 2017). These effects have been found in teachers, too (Bradshaw et al., 2008; Waters, 2011; Ross et al., 2012).

Overall, our findings suggest the need for policies and interventions aimed to promote positive emotions at school. At the same time, positive emotions should not be considered as the final aim of interventions in the workplace. Current literature suggests that happiness itself does not promote positive health outcomes in the long term (Lyubomirsky, 2012). Organizational changes are needed to pursue an effective health promotion. International surveys showed that teachers are at a high risk of developing illness and burnout disorders, other than attrition, low self-efficacy, and dissatisfaction (OECD, 2014). While the most recent models regarding the effect of positive emotions on health outcomes showed the buffering role of positive affect in stress conditions (Pressman et al., 2018), international surveys show that teachers at a lower risk for burnout and dissatisfaction receive positive, valuable feedback from colleagues and mentors, have a satisfied supervisor, and feel they can improve students’ ways of learning and thinking (OECD, 2014). These data suggest that positive interventions could strengthen positive organizational dimensions and teachers’ psychological resources, promoting teachers’ healthy behaviors and conditions.

At the same time, the study is not without limitations. Firstly, this is a cross-sectional study, thus it is highly exposed to autoregressive biases (Maxwell and Cole, 2007). Secondly, the composition of our sample does not allow us to consider differences among teachers working in different school levels, despite several studies (e.g., OECD, 2014; Ainley and Carstens, 2018), showed that teachers are differentially exposed to risk and protective factors according to their school level. At the same time, while the high percentage of women in the sample reflects the general feminization of this profession (Drudy, 2008), this may have influenced our results, as suggested by previous studies on gender differences in health prevention and intervention (Gyllensten and Palmer, 2005; Hiller et al., 2017).

## Data Availability Statement

The datasets generated for this study are available on request to the corresponding author.

## Ethics Statement

The study involving human participants were reviewed and approved by the Scientific Board of LUMSA University, Rome. The participants provided their written informed consent to participate in this study.

## Author Contributions

PB, SD, and CF designed and carried out the study, contributed to the analysis of the results and to the writing of the manuscript. IB, BR, JB, and DB collected the data, and contributed to the analysis of the results and to the writing of the manuscript. PB, SD, and DB supervised the study design and the manuscript draft.

## Conflict of Interest

The authors declare that the research was conducted in the absence of any commercial or financial relationships that could be construed as a potential conflict of interest.
